# A non-invasive, patient-derived organoid platform from uterine lavage fluid for modeling endometrial implantation and disease

**DOI:** 10.1186/s13287-026-05166-3

**Published:** 2026-07-14

**Authors:** Huiyu Qiu, Zhiwen Fan, Di Liu, Li Deng, Xiaoxia Wang, Yuanyuan Yang, Tianli Yang, Fen Tian, Jie Hao, Qiong Zhang, Bin Xu, Jing Fu, Huiling Ni, Yinshuang Li, Hankun Su, Fen Xiao, Yuanyuan Sun, Jingjing Chen, Zhiying Duan, Jing Zhao, Hui Li, Yanping Li

**Affiliations:** 1 Reproductive Medicine Center, Xiangya Hospital of Central South University, Changsha, Hunan China; 2 Clinical Research Center for Women’s Reproductive Health in Hunan Province, Changsha, Hunan China; 3 Department of Gynecology, The Third Xiangya Hospital of Central South University, Changsha, Hunan China; 4 Department of Obstetrics and Gynecology, Center for Reproductive Medicine, International School of Medicine, The Fourth Affiliated Hospital of School of Medicine, International Institutes of Medicine, Zhejiang University, Yiwu, Zhejiang China; 5 Center for Medical Genetics, School of Life Sciences & Hunan Key Laboratory of Molecular Precision Medicine, Department of Oncology, Xiangya Hospital of Central South University, Changsha, Hunan China; 6 Hunan Key Laboratory of Molecular Precision Medicine, Department of Oncology, Xiangya Hospital of Central South University, Changsha, Hunan China; 7 Department of Metabolism and Endocrinology, The Second Xiangya Hospital of Central South University, Changsha, Hunan China

**Keywords:** Endometrial organoid, Uterine lavage fluid, Non-invasive, Intrauterine adhesion, Embryo-epithelium interaction

## Abstract

**Background:**

Endometrial organoids, with their three-dimensional structure and robust hormonal responsiveness, have emerged as valuable models for investigating uterine physiology and embryo implantation. However, conventional derivation from endometrial biopsies is invasive, carries surgical risks, and limits repeated sampling. This study introduces uterine lavage fluid as a non-invasive source for generating endometrial organoids.

**Methods:**

We established endometrial organoids from uterine lavage fluid (UF-EOs) and compared them with matched tissue-derived endometrial organoids (T-EOs) for morphology, proliferation, epithelial marker expression, transcriptomic profiles, and hormone responsiveness. Lavage fluid was collected from reproductive-age women with normal endometrium and from patients with intrauterine adhesions (IUA). Differential gene-expression analysis between IUA-derived and control organoids was performed to identify IUA-associated signatures. A microinjection platform was developed to deliver HTR8/SVneo trophoblast spheroids into the organoid lumen.

**Results:**

UF-EOs were successfully derived from 90% of control individuals (9/10) and 60% of IUA patients (6/10). UF-EOs and T-EOs were morphologically indistinguishable, with comparable proliferation rates (104.6 ± 6.21 versus 106.4 ± 5.16; *P* > 0.05) and equivalent expression of epithelial markers (EpCAM, Cytokeratin 7, E-cadherin). Transcriptomic analysis showed tight paired clustering of UF-EOs with matched T-EOs across three independent patients. Both organoid types demonstrated equivalent hormone responsiveness, with progesterone-induced secretory differentiation evidenced by glandular folding, subnuclear vacuolization, glycogen accumulation, and upregulation of SPP1, PAEP, and 17HSDβ2. IUA-derived UF-EOs displayed 190 differentially expressed genes, with enrichment of cell cycle, TGF-β, and Hippo signaling pathways. TGF-β2 and Smad2 were significantly upregulated and SOX9, PCNA, and Ki67 were downregulated, consistent with compromised regenerative capacity. HTR8/SVneo spheroids were successfully microinjected into organoid lumens, with luminal localization observed at 24 h.

**Conclusion:**

We have introduced uterine lavage fluid as a feasible source for generating endometrial organoids with equivalent fidelity to tissue-derived counterparts. UF-EOs provide a non-invasive alternative that enables organoid-based research in populations previously excluded from invasive sampling, including patients with intrauterine adhesions. Our findings demonstrate applications in disease mechanism investigation, endometrial receptivity assessment, and modeling of early embryo-epithelium interactions. Future studies employing larger cohorts and integrated multicellular systems will be essential to validate the clinical utility of this approach.

**Graphical Abstract:**

**Figure:**
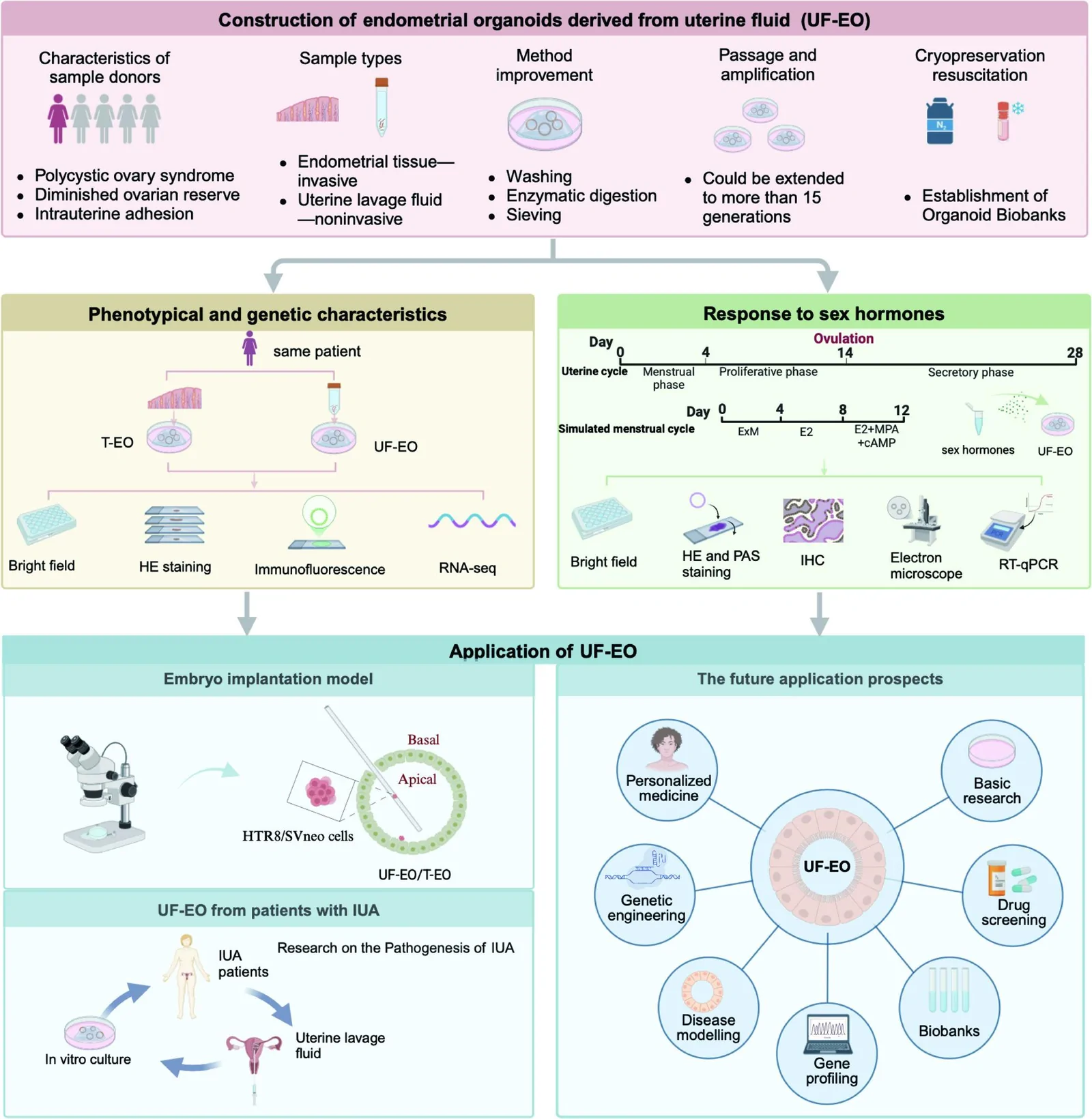

**Supplementary Information:**

The online version contains supplementary material available at https://doi.org/10.1186/s13287-026-05166-3.

## Introduction

The endometrium is a uniquely dynamic tissue that undergoes cyclic remodeling to enable embryo implantation and establish pregnancy [1]. Understanding the molecular dialogues at the maternal-fetal interface necessitates experimental models that recapitulate in vivo architecture and function [2]. Endometrial organoids have emerged as transformative three-dimensional model systems, capable of long-term expansion, genomic stability, and hormonal responsiveness, thereby enabling studies of endometrial biology, disease pathogenesis, and therapeutic screening [3–7].

However, conventional organoid derivation relies on invasive endometrial biopsy or hysterectomy specimens [3, 4], limiting repeated sampling and excluding patients with contraindications to tissue acquisition. A significant advance was achieved with the derivation of organoids from menstrual fluid, offering a non-invasive alternative for a subset of individuals [8–10]. Nevertheless, this approach is inherently restricted to menstruating individuals, excluding key patient cohorts including those with amenorrhea, anovulation, or postmenopausal status [11–14]. Unlike menstrual fluid, uterine lavage can be performed independently of cycle phase or hormonal status. Consequently, there remains an unmet need for a universally applicable, non-invasive method of generating endometrial organoids that does not depend on a functional menstrual cycle or cause endometrial trauma.

Inspired by advances in generating patient-derived organoids from other bodily fluids, such as urine [15], we hypothesized that uterine lavage fluid could serve as an alternative, universally accessible source of endometrial cells. Prior studies have confirmed the presence of viable endometrial cells in uterine cavity aspirates [16, 17], supporting the biological plausibility of this approach. Here, we report the establishment and comprehensive characterization of endometrial organoids derived from uterine lavage fluid (UF-EOs), demonstrating their equivalence to tissue-derived organoids (T-EOs) in morphology, proliferation, hormone responsiveness, and transcriptomic profile.

We demonstrate that UF-EOs can be generated from a broad patient spectrum, including individuals with polycystic ovary syndrome (PCOS), recurrent implantation failure (RIF), and intrauterine adhesions (IUA). Notably, for patients with IUA, where endometrial scarring and cavity distortion [18, 19] render conventional biopsy technically challenging or contraindicated, uterine lavage offers a safe, repeatable alternative for obtaining patient-specific tissue. This capability addresses a gap in IUA research, where the lack of patient-specific models has limited mechanistic understanding and therapeutic development.

Beyond derivation, we sought to establish UF-EOs as a functional model for studying embryo-epithelium interactions. To this end, we developed a microinjection-based approach to introduce trophoblast spheroids into the luminal compartment of UF-EOs, enabling the study of early embryo-epithelium interactions in a three-dimensional, patient-derived context.

Collectively, this work introduces uterine lavage fluid as a source for generating patient-specific endometrial organoids. The UF-EO platform enables investigation of endometrial physiology, including hormone responsiveness and early embryo-epithelium interactions, and provides a model system for studying gynecological disorders such as IUA.

## Materials and methods

### Study participants and ethics

The research was conducted in accordance with the Declaration of Helsinki and was carried out at the Department of Reproductive Medicine, Xiangya Hospital, Central South University, following approval from the Medical Ethics Committee of Xiangya Hospital (registration no. 2022007). All patients signed written informed consent. Specimens were excluded if clinical or pathological diagnoses indicated endometrial disorders such as endometriosis, adenomyosis, or endometrial cancer, unless specifically enrolled for IUA studies. Twenty patients were enrolled: ten consented to provide paired samples of uterine lavage fluid and endometrial biopsy, and ten diagnosed with intrauterine adhesions (IUA) underwent only uterine lavage fluid aspiration, due to technical difficulty and safety concerns associated with biopsy in scarred or distorted cavities. The clinical characteristics of the patients are detailed in Tables 1 and 2.

**Table 1:** Clinical characteristics of the control group

Sample ID	Previous obstetric history	Age	Clinical diagnosis	Treatment	Drug protocol	Outcome
Con 1	G0	27	Primary infertility; Tubal factor	FET single blastocyst, Day 5	HRT	Intrauterine pregnancy
Con 2	G3P1A2	31	Secondary infertility; Male factors	FET two blastocysts, Day 5	HRT	Full term delivery
Con 3	G0	34	Primary infertility; RIF; DOR; Male factors	FET single cleavage stage embryo, Day 3	HRT	Intrauterine pregnancy
Con 4	G0	27	Primary infertility; Tubal factor; RIF; Male factors	FET single blastocyst, Day 5	HRT	Intrauterine pregnancy
Con 5	G2P1A1	45	Secondary infertility; Tubal factor; DOR	FET single blastocyst, Day 5	Natural cycle	Full term delivery
Con 6	G1P1	37	Secondary infertility; Tubal factor	FET single blastocyst, Day 5	HRT	Intrauterine pregnancy
Con 7	G0	25	Primary infertility; Tubal factor; DOR	FET single blastocyst, Day 5	HRT	Biochemical pregnancy
Con 8	G1P0A1	29	Secondary infertility; Tubal factor; PCOS	FET single blastocyst, Day 5	HRT	Intrauterine pregnancy
Con 9	G1P1	33	Secondary infertility; PID	FET single blastocyst, Day 5	Natural cycle	Not pregnant
Con 10	G2P2	39	Secondary infertility; PID	FET single cleavage stage embryo, Day 3	Natural cycle	Not pregnant

G: Gestation; P: Parturition; A: Abortion; RIF: Repeated implant failure; DOR: Diminished ovarian reserve; PID: Pelvic inflammatory disease; PCOS: Polycystic ovary syndrome

**Table 2:** Clinical characteristics of the group with intrauterine adhesions (IUA)

Sample ID	Previous obstetric history	Age	Menstrual flow volume changes	Clinical diagnosis	AFS scores	Results of organoid culture
IUA 1	G4P1A3	39	Reduce by one-third	Secondary infertility; IUA	10	Cultured to the fourth passage
IUA 2	G1P1	37	Unchanged	Secondary infertility; Tubal factor; IUA	8	Cultured to the fourth passage
IUA 3	G3P1A2	36	Reduce by one-third	Secondary infertility; IUA	10	Culture failure
IUA 4	G1P0A1	32	Unchanged	Secondary infertility; IUA	8	Cultured to the third passage
IUA 5	G4P2A2	35	Reduce by one-third	Secondary infertility; IUA	8	Culture failure
IUA 6	G4P2A2	33	Unchanged	Secondary infertility; IUA	8	Culture failure
IUA 7	G2P1A1	34	Reduce by two-third	Secondary infertility; IUA	8	Cultured to the second passage
IUA 8	G0P0	38	Reduce by two-third	Primary infertility; IUA	10	Culture failure
IUA 9	G2P0A2	36	Reduce by one-third	Secondary infertility; IUA	8	Cultured to the third passage
IUA 10	G4P1A3	38	Unchanged	Secondary infertility; IUA	8	Cultured to the third passage

One additional patient (IUA-P, 33 years, AFS score 3) was included in the pilot study and successfully cultured to passage 4. Data from this patient were not included in the main analysis

G: Gestation; P: Parturition; A: Abortion; IUA: intrauterine adhesion

### Sampling methods of uterine lavage fluid and endometrial tissue

#### Uterine lavage fluid collection

A vaginal speculum was inserted to visualize the cervix, which was cleansed with sterile saline. A sterile artificial insemination catheter (HengHao Technique, HH61147) was introduced through the cervical canal into the uterine cavity and in the mid-cavity region, approximately 1–2 cm from the fundus in a norm-sized uterus (adjusted according to uterine depth measured during catheter insertion). One milliliter of sterile saline (37 °C) was gently infused via a 2.5 ml syringe attached to the catheter. Following a 30-second dwell time, gentle manual suction (approximately 2–3 ml negative pressure) was applied to retrieve the fluid. The mean recovery volume was recorded, and samples with < 0.3 ml return were excluded due to insufficient cell yield. The catheter was withdrawn, and the external surface was wiped with sterile gauze to minimize cervical mucus contamination. Aspirated samples were immediately transferred to sterile 5 ml centrifuge tubes and stored at 4 °C. Time from collection to laboratory processing was recorded for each sample. Specimens exceeding 4 h were excluded based on preliminary viability assessments (data not shown).

#### Endometrial tissue biopsy

Immediately following lavage fluid collection, endometrial tissue was obtained using a disposable suction curettage tube (Jinbeikang, GXGD). Tissue fragments (approximately 0.5 cm³) were collected and placed in 1 ml sterile phosphate-buffered saline (PBS, Gibco, 10010023) supplemented with 1% penicillin-streptomycin (Gibco, 15140122) in a sterile 2 ml microcentrifuge tube. Samples were stored at 4 °C. Time from collection to laboratory processing was recorded. Specimens exceeding 4 h were excluded.

#### Quality control

Samples were excluded based on quality criteria: uterine lavage fluid exhibiting hematocrit-like appearance or visible clot formation, and endometrial tissue showing necrotic characteristics (discolored, friable, or non-elastic).

### Culture of tissue-derived endometrial organoids (T-EOs)

#### Tissue processing and enzymatic dissociation

The endometrial tissue was transferred to a sterile six-well plate, washed with PBS, and cut into 1 mm³ fragments using scissors. The tissue fragments were then transferred to a new 15 ml centrifuge tube. Tissue samples were digested with 2 mg/ml collagenase IV (Solarbio, C8160) in DMEM/F12 (Gibco, 21041025) at 37 °C for 30 min with intermittent mechanical trituration (every 10 min, 10–15 pipetting strokes). The digestion mixture was centrifuged at 800 × g for 5 min at 4 °C. The supernatant was aspirated, and the cellular pellet was resuspended in 10 ml PBS.

#### Glandular isolation

The cell suspension was filtered through a 100 μm cell strainer (Biosharp, BS-100-XBS) placed over a 50 ml centrifuge tube. The strainer was inverted over a 10 cm Petri dish, and 5 ml PBS was applied to the reverse surface to backwash and collect retained glandular structures. Glandular elements were transferred to a 15 ml tube and centrifuged at 800 × g for 5 min.

#### Single-cell dissociation

The glandular pellet was resuspended in 1 ml TrypLE Express Enzyme (1X, phenol red-free) (Gibco, 12604013) and incubated at 37 °C for 5–10 min with gentle agitation (every 2–3 min). Digestion was terminated by adding DMEM/F12 medium. The suspension was centrifuged at 800 × g for 5 min.

#### Matrigel embedding and culture

The resulting pellet was resuspended in Matrigel (Corning, 356231) at a final concentration of 80% (mixed 4:1 with ice-cold organoid basal medium). Fifty-microliter droplets of the Matrigel-cell suspension were dispensed into the centers of pre-warmed 24-well plates (Costar, 3524). Plates were incubated at 37 °C with 5% CO₂ for 20 min to allow Matrigel polymerization. Organoids were overlaid with 500 µl expansion medium (ExM) per well. Medium was refreshed every 2–3 days. Cultures were passaged every 7–10 days using TrypLE Express for 5 min at 37 °C followed by mechanical dissociation.

#### Expansion medium composition

Expansion medium (ExM) was prepared in Advanced DMEM/F12 (Gibco, 21041025) supplemented with N2 (1X; Gibco, 17502048), B27 minus vitamin A (1X; Gibco, 12587010), penicillin-streptomycin (1X; Gibco, 15140122), 1.25 mM N-acetyl-L-cysteine (Sigma-Aldrich, A7250), 2 mM L-glutamine (Gibco, 25030081), 10 mM nicotinamide (Sigma-Aldrich, 73240), 50 ng/ml recombinant human EGF (PeproTech, 100 − 15), 100 ng/ml recombinant human Noggin (PeproTech, 120–10 C), 500 ng/ml recombinant human R-spondin-1 (R&D Systems, 4645-RS), 100 ng/ml recombinant human FGF-10 (PeproTech, 100 − 26), 50 ng/ml recombinant human HGF (PeproTech, 100 − 39), and 500 nM A83-01 (MedChemExpress, HY-10432).

### Culture of uterine lavage fluid-derived endometrial organoids (UF-EOs)

#### Cell and glandular debris collection

Uterine lavage fluid samples were centrifuged at 800 × g for 5 min. The supernatant was aspirated, and the pellet containing cells and glandular debris was resuspended in DMEM/F12 medium. Optional red blood cell lysis using ACK lysis buffer was performed if gross blood contamination was observed.

#### Mechanical dissociation and optional enzymatic digestion

The suspension was gently pipetted 10–15 times using a 1 ml wide-bore pipette tip to mechanically separate glandular debris. Larger endometrial fragments, if present, were subjected to enzymatic digestion with TrypLE Express Enzyme (1X) at 37 °C for 5–10 min with gentle agitation every 5 min. Digestion was terminated by adding three volumes of DMEM/F12 medium.

#### Final pellet preparation

The suspension was centrifuged at 800 × g for 5 min. The supernatant was aspirated, and the pellet was washed once with 10 ml PBS. Following an additional centrifugation at 800 × g for 5 min, the final pellet was resuspended in Matrigel (Corning, 356231) at a final concentration of 80% (mixed 4:1 with ice-cold organoid basal medium).

#### Matrigel embedding and culture

Fifty-microliter droplets of the Matrigel-cell suspension were dispensed into the center of individual wells of a pre-warmed 24-well plate (Costar, 3524). Plates were incubated at 37 °C with 5% CO₂ for 20 min. Organoids were overlaid with 500 µl expansion medium (ExM) per well. Medium was refreshed every 2–3 days. Cultures were passaged every 7–10 days using TrypLE Express for 5 min at 37 °C followed by gentle mechanical dissociation. Both T-EOs and UF-EOs were processed in parallel using the same batch of Matrigel and culture media to minimize batch effects.

### Freezing and recovery of organoids

#### Matrigel dissolution

Culture medium was aspirated from each well, and 500 µl Cell Recovery Solution (Corning, 354253) was added. The plate was incubated on ice for 20 min with gentle pipetting to facilitate Matrigel dissolution. The contents were collected into low-binding microcentrifuge tubes (Eppendorf, 0030108116) using low-retention pipette tips.

#### Organoid harvesting and washing

Tubes were centrifuged at 600 × g for 5 min at 4 °C to pellet organoids. The supernatant was aspirated, and organoids were resuspended in 10 ml ice-cold DMEM/F12 (Gibco, 21041025). The suspension was gently pipetted 20–30 times with a 5 ml serological pipette to partially disrupt Matrigel remnants without dissociating organoid structures. Following centrifugation at 600 × g for 5 min at 4 °C, the supernatant was aspirated.

#### Cryopreservation

The pellet was resuspended in 0.8–1.0 ml freezing medium consisting of 90% fetal bovine serum (FBS) and 10% dimethyl sulfoxide (DMSO; Sigma-Aldrich, D2650). The suspension was gently mixed and transferred to labeled cryovials (Corning, 430659). Cryovials were immediately placed in a controlled-rate freezing container (Mr. Frosty, Thermo Fisher Scientific, 5100-0001) at − 80 °C overnight, then transferred to liquid nitrogen vapor phase for long-term storage.

#### Thawing and recovery

Cryovials were rapidly thawed in a 37 °C water bath with gentle agitation for 1–2 min until only a small ice crystal remained. The freezing medium was rapidly diluted into 10 volumes of pre-warmed DMEM/F12. Organoids were pelleted at 300 × g for 5 min, washed once with DMEM/F12 supplemented with 10 µM Y-27632 (ROCK inhibitor; Selleck, S1049), and resuspended in Matrigel (Corning, 356231) at a final concentration of 80% (mixed 4:1 with ice-cold organoid basal medium). Fifty-microliter droplets were dispensed into pre-warmed 24-well plates and cultured in expansion medium supplemented with 10 µM Y-27632 for the first 48 h to enhance post-thaw recovery.

### Organoid formation efficiency from single cells and CellTiter-Lumi™ Luminescent 3D viability assay

UF-EOs and T-EOs derived from the same patient were maintained in expansion medium. Experiments were performed using organoids between passages 4 and 8, with matched passage numbers for UF-EOs and T-EOs from each individual patient. Organoids were harvested from Matrigel using Cell Recovery Solution (Corning, 354253) as described above, mechanically dissociated by vigorous pipetting, and enzymatically digested with TrypLE Express Enzyme (1X, phenol red-free; Gibco, 12604013) at 37 °C with gentle agitation every 2 min until organoids were fully dissociated (typically 5–10 min). Digestion was terminated by adding three volumes of DMEM/F12. The resulting cell suspension was filtered through a 40 μm cell strainer (Biosharp, BS-40-XBS) to obtain a single-cell suspension. Cell viability and concentration were determined by trypan blue exclusion using a hemocytometer. For organoid formation assays, 5,000 viable single cells were seeded per 20 µl Matrigel droplet in a 48-well plate (Corning, 3548) with three replicates per condition. The number of organoids formed after 10 days was scored. Cell viability was assessed using the CellTiter-Lumi™ Luminescent 3D viability assay (Beyotime, C0061S) following the manufacturer’s instructions. Briefly, the CellTiter-Lumi™ reagent was equilibrated to room temperature for at least 30 min. An equal volume of CellTiter-Lumi™ reagent was added directly to each well (1:1 ratio with existing culture medium). Following 30-minute incubation at 37 °C with gentle orbital shaking (300 rpm, 3 mm amplitude), luminescence was measured on the PerkinElmer Envision.

### RNA sequencing and bioinformatic analysis

#### Library preparation and sequencing

RNA sequencing of endometrial organoids was conducted by Novogene Co. (Changsha, China). Total RNA was extracted using the Ultra-Pure Total RNA Extracting Kit (Simgen, 5003050) following manufacturer’s instructions. RNA integrity was assessed using the RNA Nano 6000 Assay Kit of the Bioanalyzer 2100 system (Agilent Technologies, CA, USA). PCR products were purified (AMPure XP system) and library quality was assessed on the Agilent Bioanalyzer 2100 system. The clustering of the index-coded samples was performed on a cBot Cluster Generation System using TruSeq PE Cluster Kit v3-cBot-HS (Illumina) according to the manufacturer’s instructions. After cluster generation, the library preparations were sequenced on an Illumina NovaSeq platform and 150 bp paired-end reads were generated.

#### Hierarchical clustering analysis

During data preprocessing, genes with FPKM values greater than 2 across all samples were retained, resulting in 8,659 genes for subsequent analysis. Principal component analysis (PCA) and two-way hierarchical clustering were then performed on the transcriptome data from three independent patient-derived paired samples (*n* = 3 patients, each contributing both UF-EOs and T-EOs) using the OmicShare online platform (http://www.omicshare.com). Representative marker genes for epithelial, proliferative, secretory, and progenitor cell populations were annotated on the heatmap.

#### Differential expression and functional enrichment analysis

Two distinct differential expression analyses were performed. For the assessment of transcriptional equivalence between UF-EOs and T-EOs (Supplementary Fig. S3), DESeq2 (version 1.30.0) was applied to raw read counts, with thresholds of false discovery rate (FDR) < 0.05 and absolute fold change > 1.5. For the comparison between IUA-derived and control UF-EOs under EPC hormonal conditions (Fig. 5B), DEGs were identified using DESeq2 (version 1.30.0) based on raw read counts, with thresholds of FDR < 0.05 and absolute fold change > 2. Volcano plots were generated and representative DEGs implicated in IUA-related pathological processes were labeled for visualization. Gene Ontology (GO) term enrichment analysis and Kyoto Encyclopedia of Genes and Genomes (KEGG) pathway enrichment analysis were conducted using the OmicShare platform (http://www.omicshare.com) with a significance threshold of FDR < 0.05.

### Hormonal treatment

To evaluate hormonal responsiveness, organoids were cultured in ExM for 4 days, then treated with 10 nM estradiol (E2; Selleck, S1709) for 4 days. Subsequently, organoids were randomized to three groups for an additional 4 days with medium refresh every 2 days: (1) 10 nM E2 (designated as Group E), (2) 10 nM E2 combined with 1 µM medroxyprogesterone acetate (MPA; Selleck, S2567) (designated as Group EP), or (3) 10 nM E2, 1 µM MPA, and 1 µM cAMP (Sigma-Aldrich, B7880) (designated as Group EPC). A hormone-free control group was included as the negative control (designated as Group NC). NC group was maintained in ExM devoid of hormones throughout the 12-day period.

### Quantitative real-time RT-PCR analysis

Total RNA was extracted using the miRNeasy Mini Kit (Qiagen, 217004) according to the manufacturer’s instructions. The quality and concentration of the RNA were assessed using the Nanodrop ND-1000 Spectrophotometer. Subsequently, 500 ng of total RNA was reverse transcribed using the PrimeScript™ RT reagent Kit (Takara, RR037A) as per the manufacturer’s guidelines. An RNA sample without reverse transcriptase was included as a control to check for genomic DNA contamination. All RT-qPCR experiments were conducted with a non-template control.

Specific PCR amplification products were detected by the fluorescent double-stranded DNA-binding dye SYBR Green. Primers are listed in Table 3. Glyceraldehyde-3-phosphate dehydrogenase (GAPDH) was used as the housekeeping gene. RT-qPCR) was performed on QuantStudio™ 3 System (Thermo Fisher Scientific). The cycling conditions were 95 °C for 60 s, followed by 40 cycles of 95 °C for 15 s, 60 °C for 30 s, and 72 °C for 40 s. Relative gene expression levels were calculated using the ΔΔCt method (Ct of the target gene minus Ct of the housekeeping gene) and were primarily compared between the ‘sample’ and ‘reference’ to express fold changes.

**Table 3:** Primer Sequence for RT-qPCR

Gene symbol	Forward primer	Reverse primer
GAPDH	GAAGGTCGGAGTCAACGGATTT	GAATTTGCCATGGGTGGAAT
SPP1	CGAGGTGATAGTGTGGTTTATGG	GCACCATTCAACTCCTCGCTTTC
17HSDβ2	TGAATGTCAGCAGCATGG	GGAAAGCTCCAGTCTCATAAC
PAEP	ATGGCGACCAACAACATC	CTCTCCAAGGACCTTCTTCT
ESR1	CCCACTCAACAGCGTGTCTC	CGTCGATTATCTGAATTTGGCCT
PGR	CGCGCTCTACCCTGCACTC	TGAATCCGGCCTCAGGTAGTT
Ki67	TCCAGACGCCAAAATAAGACTG	TCCATCTCTGGGGAGGTCTTC
LRIG1	CTGGACGCGGAGCCTAAAC	TGTAGGTTCGGCAAGTCCTCA
PROM1	AGTCGGAAACTGGCAGATAGC	GGTAGTGTTGTACTGGGCCAAT
PCNA	CCTGCTGGGATATTAGCTCCA	CAGCGGTAGGTGTCGAAGC
TOP2A	ACCATTGCAGCCTGTAAATGA	GGGCGGAGCAAAATATGTTCC
CDH1	CGAGAGCTACACGTTCACGG	GGGTGTCGAGGGAAAAATAGG
KRT7	TCCGCGAGGTCACCATTAAC	GCTCTGTCAACTCCGTCTCAT
EPCAM	AATCGTCAATGCCAGTGTACTT	TCTCATCGCAGTCAGGATCATAA
MUC1	TGCCGCCGAAAGAACTACG	TGGGGTACTCGCTCATAGGAT
MUC20	ATGACAACGGACGACACAGAA	TCAGCGTTTGAGTTTCCAGAG
TGF-β1	GACTCGCCAGAGTGGTTATCT	CGGTAGTGAACCCGTTGAT
TGF-β2	CAGCACACTCGATATGGACCA	CCTCGGGCTCAGGATAGTCT
Smad2	GGGTTTTGAAGCCGTCTATCAGC	CCAACCACTGTAGAGGTCCATTC
COL1A1	GATTCCCTGGACCTAAAGGTGC	AGCCTCTCCATCTTTGCCAGCA
CTGF	CTTGCGAAGCTGACCTGGAAGA	CCGTCGGTACATACTCCACAGA

### Immunohistochemistry (IHC)

UF-EOs and T-EOs were dissociated from Matrigel using the Cell Recovery Solution and then fixed with 4% paraformaldehyde. They were subsequently encapsulated into Histogel (Epredia, HG-4000-012) and 1% agarose (Melford, MB1200), followed by dehydration and embedding in paraffin wax. Tissue sections of 4 μm were cut from formalin-fixed paraffin-embedded UF-EOs and T-EOs. These sections were dewaxed, rehydrated, and boiled in Tris-EDTA buffer (ZSGB, ZLI-9071) for 15 min in a microwavable pressure cooker. The tissue sections were incubated in Endogenous Peroxidase Blocking Buffer (Beyotime, P0100A) for 15 min to inhibit endogenous peroxidase activity. They were then blocked for 1 h in 3% BSA solution to prevent non-specific antibody binding. The sections were incubated overnight at 4 °C with the appropriate primary antibodies: PAEP (Abcam, ab53289, 1:500). An HRP-conjugated secondary antibody (ZSGB, PV-8000) was used, followed by DAB substrate (Sigma-Aldrich, D4168). The sections were then lightly counterstained with hematoxylin, before being dehydrated and mounted.

### Immunofluorescence (IF)

Sections of UF-EOs and T-EOs were dewaxed, rehydrated, and boiled in Tris-EDTA buffer in a microwavable pressure cooker for 5 min. They were then blocked for 1 h in a solution containing 3% BSA and 0.3% Triton X-100. Following this, the sections were incubated overnight at 4 °C with the appropriate primary antibodies: EPCAM (Cell Signaling Technology, 2929, 1:100), E-cadherin (Cell Signaling Technology, 3195, 1:100), and Cytokeratin 7 (Dako, M7018, TE, 1:200). The next day, the sections were washed three times for 15 min each in PBS. They were incubated for 1 h at room temperature in PBS with secondary antibodies: Alexa Fluor 568 goat anti-rabbit IgG(H + L) (Invitrogen, A11011), CoraLite488-conjugated Affinipure Goat Anti-Mouse IgG (H + L) (Proteintech, SA00013-1), or CoraLite488-conjugated Affinipure Goat Anti-Rabbit IgG (H + L) (Proteintech, SA00013-2), all at 1:500, along with DAPI (Invitrogen, S36964). Finally, the sections were washed in PBS three times and mounted.

### Hematoxylin and eosin (H&E) staining

Sections of UF-EOs and T-EOs were dewaxed, rehydrated, and stained in hematoxylin for 10 min. They were then washed in running water for 20 min and stained in eosin for 5 min, followed by dehydration and mounting.

### Periodic acid Schiff (PAS) staining

Sections of UF-EOs and T-EOs were dewaxed, rehydrated, and treated with 1% periodic acid (Sigma-Aldrich, 395132) for 15 min. The sections were then rinsed in water, immersed in Schiff’s reagent (Sigma-Aldrich, 3952016) for 15 min, and subsequently rinsed in water again. They were counterstained with hematoxylin for 2 min, washed in running tap water, dehydrated, and mounted.

### Transmission electron microscopy (TEM)

T-EOs and UF-EOs from the EPC group were initially fixed in 2.5% glutaraldehyde for a minimum of six hours and were subsequently stored at 4 °C. The samples underwent four rinses with 0.1 M phosphate buffer, each lasting ten minutes, followed by post-fixation in 1% osmium tetroxide for 1.5 to 2 h. The dehydration process was executed sequentially using ethanol concentrations of 50%, 70%, and 90%, followed by a 20-minute treatment with a 1:1 mixture of 90% ethanol and acetone. This was succeeded by three changes of 100% acetone, each lasting 15 to 20 min. Initial embedding was conducted in a 2:1 mixture of acetone and embedding medium at room temperature for 3 to 4 h, transitioning to a 1:2 ratio overnight. Final embedding was performed in pure embedding medium at 37 °C for 2 to 3 h. Polymerization was undertaken at 37 °C overnight, followed by 45 °C for 12 h, and 60 °C for 48 h. Ultrathin sections, approximately 70 nm in thickness, were prepared using an ultramicrotome and subsequently stained with 3% uranyl acetate and lead citrate. The samples were analyzed using a transmission electron microscope, and electron micrographs were captured to document the ultrastructural characteristics of the organoids.

### In Vitro Embryo Implantation Model Construction

#### Fluorescent labeling

HTR8/SVneo cells were transduced with lentiviral vectors encoding GFP or mCherry, followed by puromycin selection (2 µg/ml) for 7 days to establish stable fluorescent cell lines. UF-EOs were dissociated into single cells, transduced with lentiviral GFP vectors, and re-embedded in Matrigel to generate fluorescent organoids. Hoechst 33,342 (Beyotime, C1029) was added to label UF-EO nuclei 30 min prior to microinjection.

#### Trophoblast spheroid formation

HTR8/SVneo cells were seeded in U-shaped ultra-low attachment 96-well plates (Corning, 7007) at a density of 800 cells per well and cultured in DMEM/F12 supplemented with 10% FBS for 24 h to facilitate spheroid formation. Spheroids with a diameter of 80–100 μm were selected for microinjection.

#### Microinjection procedure

GFP-labeled UF-EOs were cultured for an additional 7–10 days after passage until they developed a central lumen with a diameter of 300–500 μm. Glass capillaries (Aibei Biotechnology, H0450) were pulled and connected to mouth pipettes (Aibei Biotechnology, H0820) for manual microinjection under a stereomicroscope. The capillary was inserted through the Matrigel dome periphery to access the luminal cavity, and HTR8/SVneo spheroids were injected into the lumen. Following injection, Matrigel was allowed to re-polymerize for 10 min at 37 °C before overlaying fresh medium.

#### Co-culture and imaging

Following microinjection, co-cultures were incubated for 24 h. Samples were imaged using an LSM 880 confocal microscope (Zeiss) and a CSU-W1 spinning disk confocal system (Nikon).

### Statistical analysis

GraphPad Prism version 8.0 software was used for statistical analysis. All experiments were conducted at least three times. Data are presented as mean ± SEM. Normality and homoscedasticity were assessed using the Shapiro-Wilk and Brown-Forsythe tests, respectively. For two-group comparisons, unpaired Student’s t-test (parametric) or Mann-Whitney U test (non-parametric) was used. For multiple group comparisons, one-way ANOVA followed by Tukey’s post-hoc test was employed. Statistical significance was defined as *P* < 0.05 (*P < 0.05, **P < 0.01, ***P < 0.001; ns, not significant).

## Results

### Successful derivation of UF-EOs from uterine lavage fluid

**Fig. 1:**
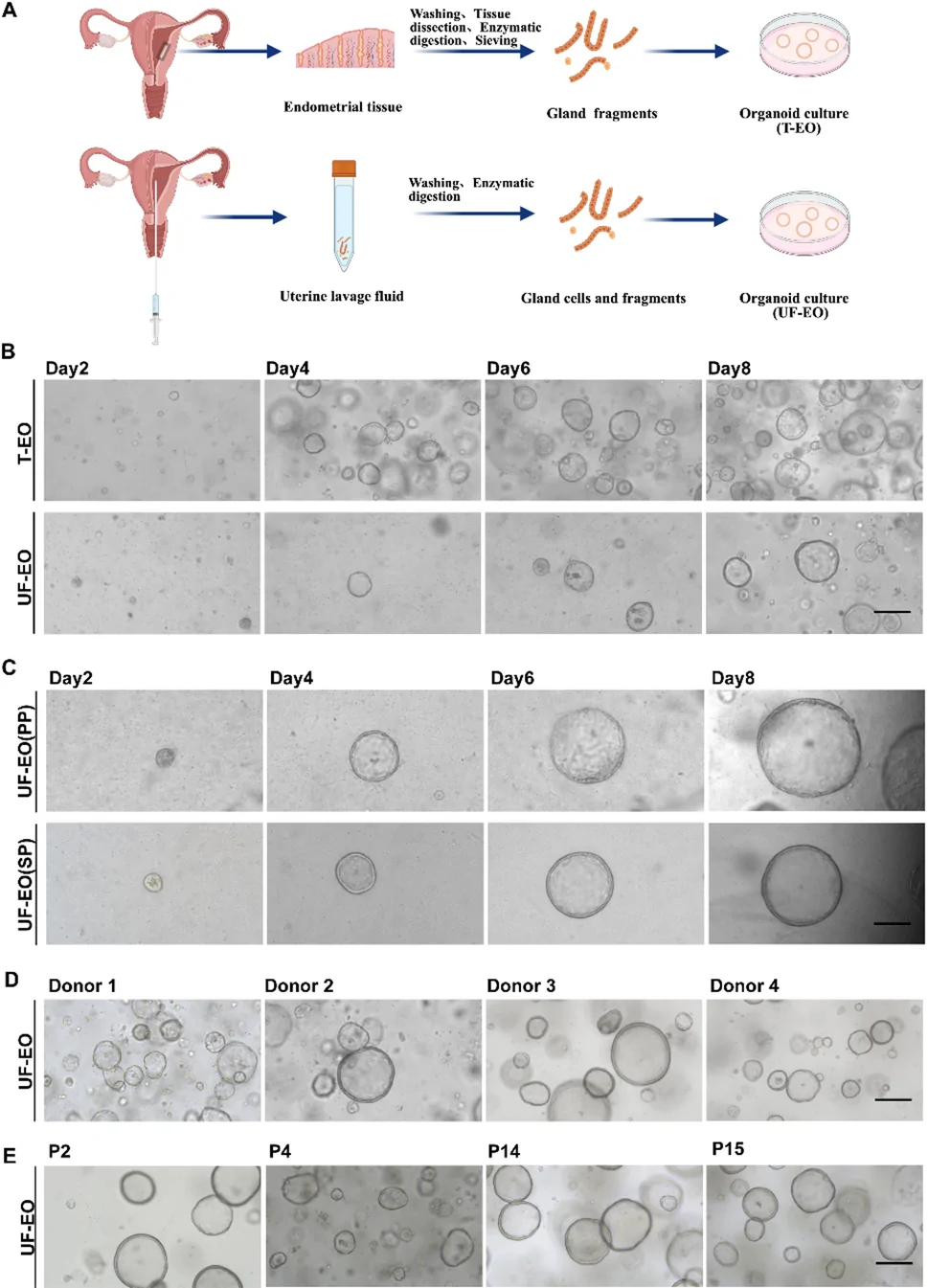
Establishment of endometrial organoids from uterine lavage fluid (UF-EOs). **A** A schematic representation depicts the derivation process of organoids from endometrial tissue and uterine lavage fluid. **B** Representative bright-field images of UF-EOs and T-EOs from matched donors at days 2–8 of culture, showing comparable cystic morphologies. Scale bar, 100 μm. **C** Representative images of UF-EOs derived from proliferative phase (PP) and secretory phase (SP) lavage fluid. Scale bar, 100 μm. **D** UF-EOs derived from four donors. Scale bar, 100 μm. **E** Different passages of organoids derived from uterine lavage fluid, showing long-term expansion and passaging capability and maintenance of morphological characteristics. Scale bar, 100 μm

We first established the feasibility of generating endometrial organoids from uterine lavage fluid (UF-EOs) (Fig. 1A). Using matched pairs of lavage fluid and endometrial tissue from the same donors, we successfully derived UF-EOs from 9 out of 10 control individuals (90% success rate). Notably, this method was also applicable to patients with IUA, yielding UF-EOs from 6 out of 10 patients with moderate-to-severe intrauterine adhesions (60% success rate) (Tables 1 and 2).

### Comparative phenotypic and genetic characterization of the UF-EOs and T-EOs

#### Morphological Properties and Long-term Expansion

**Fig. 2:**
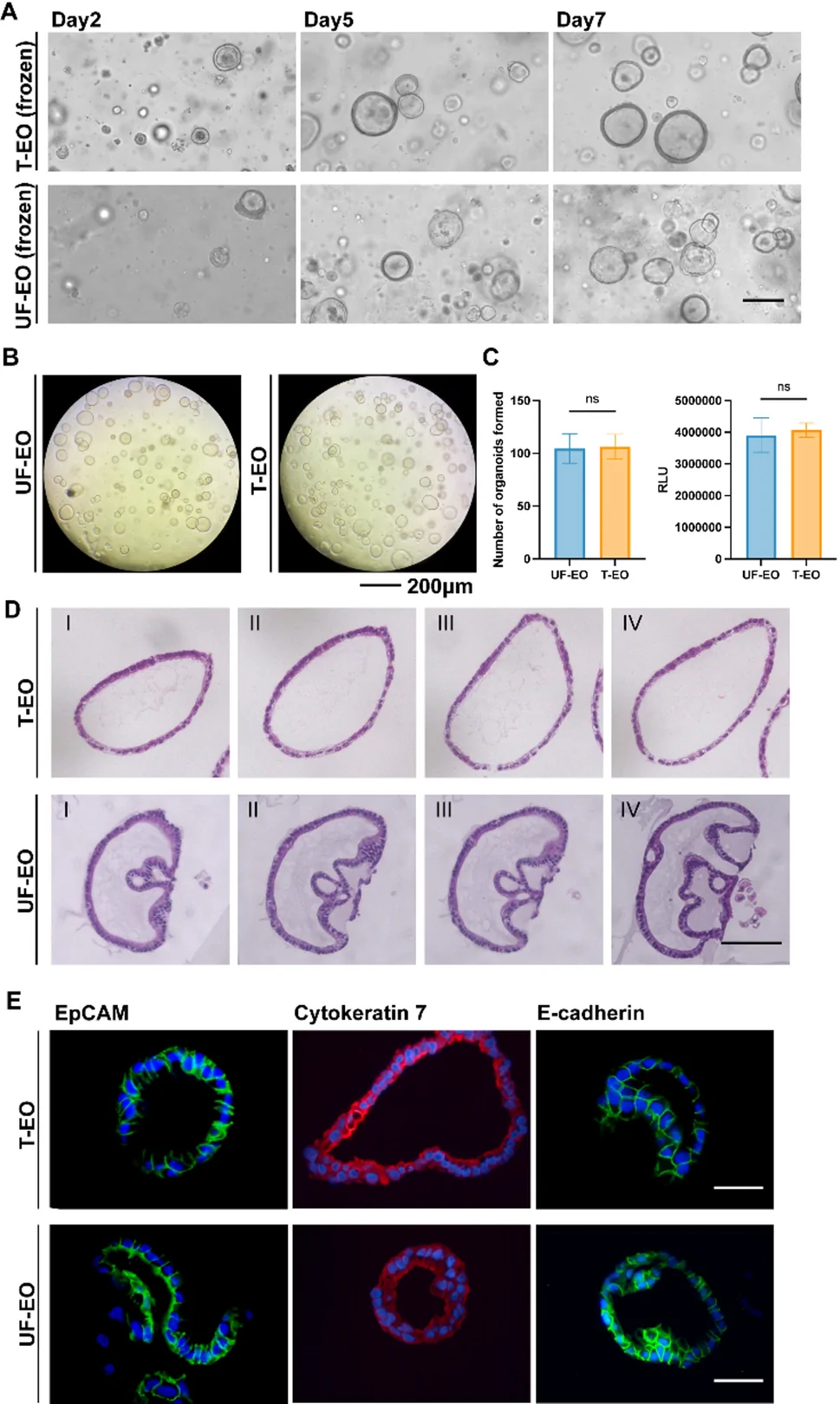
Phenotypic and functional characterization of UF-EOs. **A** Cryopreserved UF-EOs could be regrown and expanded. Scale bars, 100 μm. **B** Single-cell organoid formation assay showing comparable efficiency between UF-EOs and T-EOs. Scale bars, 200 μm. **C** Quantification of organoid formation efficiency (left) and CellTiter-Lumi™ Luminescent 3D viability assay (right) (*n* = 3 biological replicates; mean ± SEM; *P* > 0.05, unpaired t-test). **D** Hematoxylin and eosin (H&E) staining of serial paraffin sections from T-EOs and UF-EOs. Scale bar, 100 μm. **E** Immunofluorescence staining of organoids for EpCAM, CK7 and E-cadherin. Scale bar, 25 μm

UF-EOs exhibited robust growth potential in vitro, with self-organization into three-dimensional structures evident within 2–3 days of culture. Over time, these organoids demonstrated progressive enlargement and maturation, and by days 6–8, UF-EOs displayed morphological characteristics closely resembling those of T-EOs, including distinct spherical outlines (Fig. 1B). We further established UF-EOs from uterine lavage fluid collected at different menstrual phases. Organoids derived from the proliferative phase (PP) and secretory phase (SP) both exhibited typical cystic morphologies (Fig. 1C), and successful derivation from multiple independent donors demonstrated reproducibility across diverse patient populations (Fig. 1D). Long-term culture experiments showed that UF-EOs could be serially passaged at a 1:2 to 1:3 ratio every 7–10 days for over 15 passages (Fig. 1E) while maintaining regrowth capacity following cryopreservation and thawing (Fig. 2A).

#### Proliferative and metabolic activity

To assess functional equivalence, we performed single-cell growth assays. Matched-patient analysis revealed indistinguishable proliferation rates between UF-EOs and T-EOs (mean ± SEM: 104.6 ± 6.21 vs. 106.4 ± 5.16, *P* > 0.05) (Fig. 2B and C, left). Consistent with these findings, CellTiter-Lumi™ luminescent 3D viability assay demonstrated comparable cell viability between the two groups (3.91 ± 0.34 vs. 4.07 ± 0.43 × 10⁶ RLU, *P* > 0.05) (Fig. 2C, right).

#### Histological and molecular validation

**Fig. 3:**
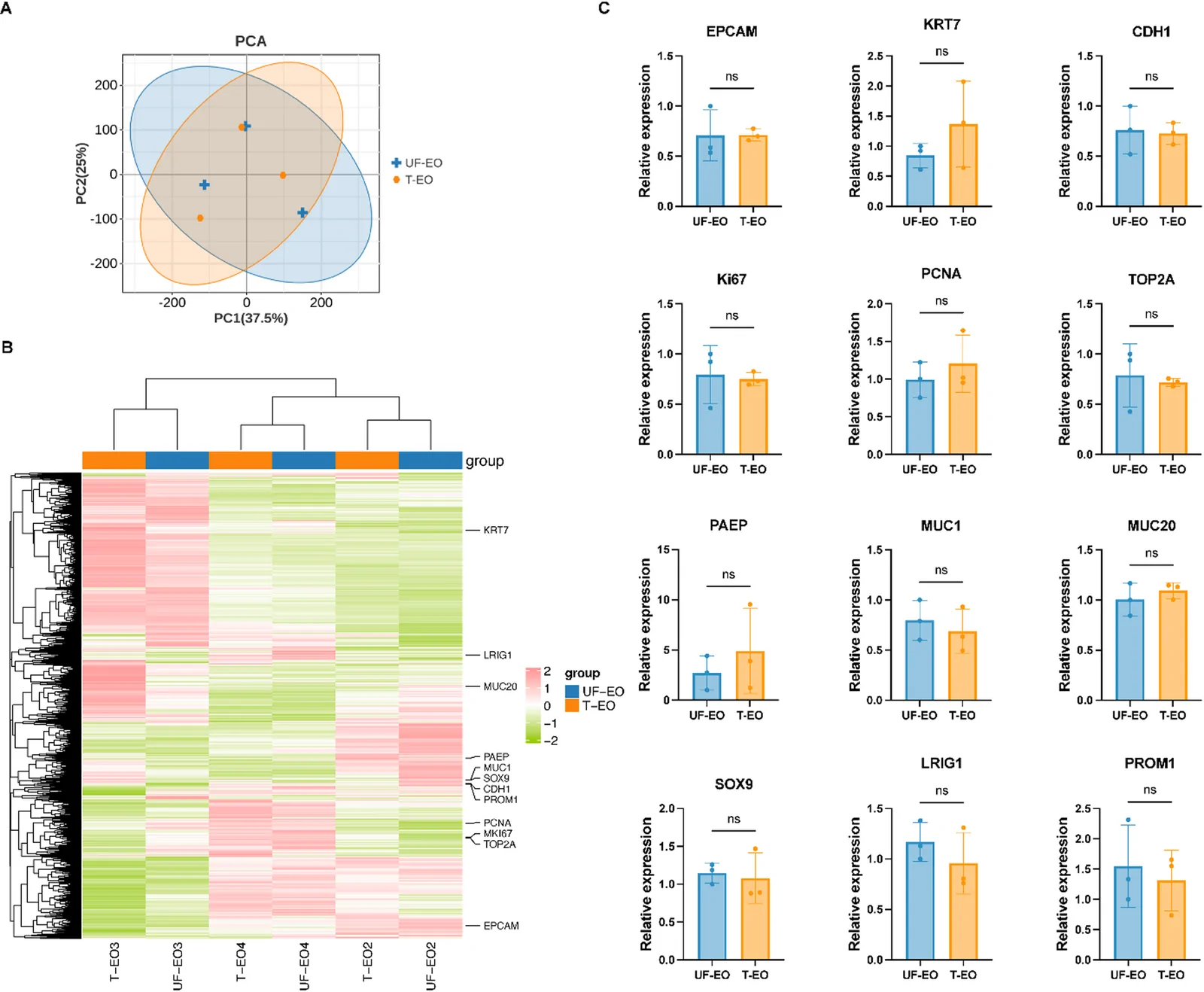
Comparative genetic characterization of UF-EOs and T-EOs. **A** Principal component analysis (PCA) of RNA sequencing data from matched UF-EOs and T-EOs based on 8,659 filtered genes. **B** A heatmap showing the pairing of UF-EOs and T-EOs within each of the three patients, based on 8,659 genes (FPKM > 2). Two-way hierarchical clustering was applied to both genes (rows) and samples (columns). Key functional gene subsets annotated on the right include epithelial (EpCAM, KRT7, CDH1), proliferation (MKI67, PCNA, TOP2A), secretory (PAEP, MUC1, MUC20), and progenitor (SOX9, LRIG1, PROM1) markers. **C** mRNA expression levels of epithelial markers (EpCAM, KRT7, CDH1), proliferation markers (Ki67, PCNA, TOP2A), secretory markers (PAEP, MUC1, MUC20), and progenitor cell markers (SOX9, LRIG1, PROM1) in UF-EOs and T-EOs

Histological and molecular analyses confirmed the endometrial identity and phenotypic fidelity of UF-EOs. Hematoxylin and eosin (H&E) staining revealed that both UF-EOs and T-EOs formed cystic gland-like structures lined by simple columnar epithelium resembling native endometrial glands (Fig. 2D). Immunofluorescence analysis revealed robust expression of canonical epithelial markers (EPCAM, CK 7, and E-cadherin) in both UF-EOs and T-EOs, with indistinguishable staining patterns between the two groups (Fig. 2E). At the transcriptomic level, principal component analysis (PCA) of RNA-seq data showed tight clustering of UF-EOs with their matched T-EO counterparts (Fig. 3A). Two-way hierarchical clustering of 8,659 genes (FPKM > 2) demonstrated paired clustering of UF-EOs with their matched T-EOs across three independent patients (Fig. 3B). To further validate the transcriptional equivalence between UF-EOs and T-EOs, differential expression analysis was performed. No significantly differentially expressed genes were identified (|fold change| > 1.5, FDR < 0.05), confirming the comparability of these two organoid sources (Supplementary Fig. S3). Quantitative PCR validation confirmed comparable expression levels of epithelial (EpCAM, KRT7, CDH1), proliferation (Ki67, PCNA, TOP2A), secretory (PAEP, MUC1, MUC20), and progenitor-associated (SOX9, LRIG1, PROM1) markers between UF-EOs and T-EOs (Fig. 3C). Collectively, these data demonstrate that UF-EOs faithfully recapitulate the architectural, functional, and molecular hallmarks of native endometrial epithelium.

### Response of organoids to sex hormones

**Fig. 4:**
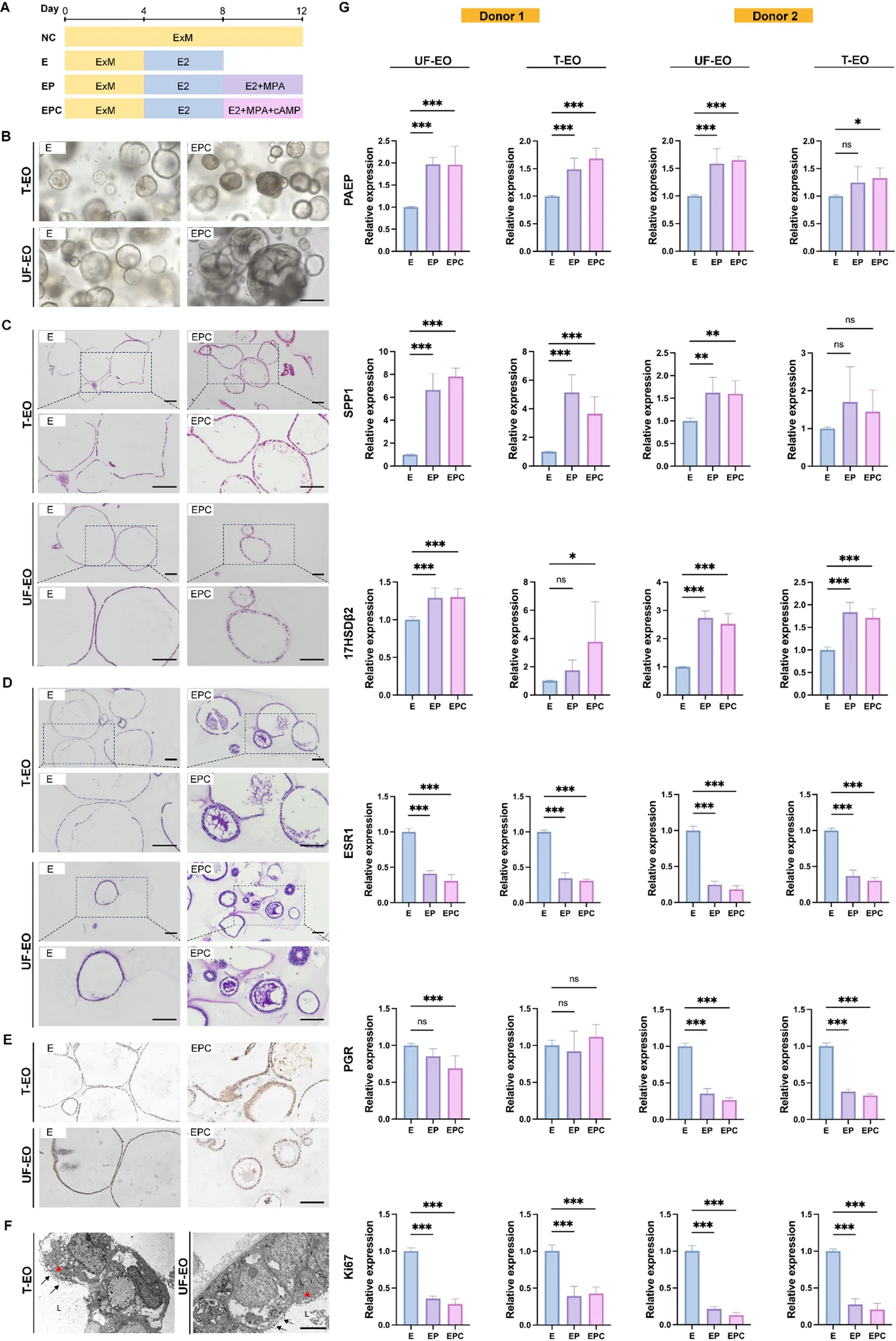
Hormone responsiveness of UF-EOs and T-EOs. **A** Protocol for hormonal stimulation of endometrial organoids. **B** Morphological changes of UF-EOs and T-EOs after hormone treatment. Scale bar, 100 μm. **C** H&E staining of UF-EOs and T-EOs after E2 or E2 + MPA+cAMP treatment. Upper panels: lower magnification views of organoid architecture. Lower panels: higher magnification views of epithelial details. Scale bar, 50 μm. **D** PAS staining of UF-EOs and T-EOs after E2 or E2 + MPA+cAMP treatment. Upper panels: lower magnification views of organoid architecture. Lower panels: higher magnification views of epithelial glycogen staining. Scale bar, 50 μm. **E** IHC for PAEP on organoids after hormonal stimulation. Scale bar, 50 μm. **F** Transmission electron microscopy (TEM) images showing the ultrastructure of T-EOs and UF-EOs from the EPC group. L, lumen; black arrows, microvilli; red arrowheads, secretory granules. Scale bar, 5 μm. **G** mRNA of markers (PAEP, SPP1, 17HSDβ2, ESR1, PGR and Ki67) was quantified using RT-qPCR in hormonally treated UF-EOs and T-EOs (from Donor 1 and Donor 2). Statistical significance was assessed using ANOVA, *P*-values are shown on each graph. Data are means ± SEMs. *P < 0.05, **P < 0.01, ***P < 0.001

We next assessed whether UF-EOs retain the dynamic hormone responsiveness characteristic of the native endometrium. Organoids were subjected to a sequential hormonal regimen mimicking the physiological menstrual cycle: expansion medium (ExM) alone (NC), estrogen (E2) alone (E), E2 plus medroxyprogesterone acetate (MPA) (EP), or E2 + MPA with cAMP (EPC) (Fig. 4A). Following progesterone exposure, both T-EOs and UF-EOs underwent morphological maturation characteristic of the secretory-phase endometrium, manifesting as increased luminal surface folding and tortuosity (Fig. 4B). H&E staining revealed subnuclear cytoplasmic vacuolization in columnar epithelial cells after E2 + MPA+cAMP treatment (right panels) compared with E2 treatment alone (left panels), with lower magnification images showing overall organoid architecture and higher magnification images detailing epithelial morphology and luminal structures (Fig. 4C). These structural changes were accompanied by glycogen accumulation, as demonstrated by PAS staining (right panels) relative to E2 alone (left panels), with lower magnification images showing organoid architecture and higher magnification images revealing epithelial glycogen accumulation (Fig. 4D). Additional representative fields of H&E and PAS staining are provided in Supplementary Fig. S4. These morphological changes occurred together with elevated expression of the glycoprotein progestagen-associated endometrial protein (PAEP), as detected by immunohistochemistry (Fig. 4E). At the ultrastructural level, TEM images of the EPC group showed apical microvilli (black arrows) and secretory granules (red arrowheads) along the luminal surface (L) in both UF-EOs and T-EOs (Fig. 4F). RT-qPCR analysis revealed that UF-EOs responded to hormonal stimulation in a manner concordant with T-EOs. Both models showed upregulation of secretory-phase markers (PAEP, SPP1, and 17HSDβ2) and concomitant downregulation of proliferation marker Ki67 and hormone receptors ESR1, with modulation of PGR expression upon progesterone exposure (Fig. 4G). These findings support UF-EOs as physiologically competent models that recapitulate key features of endometrial cycle transitions.

### UF-EOs as a tool for intrauterine adhesion pathogenesis

**Fig. 5:**
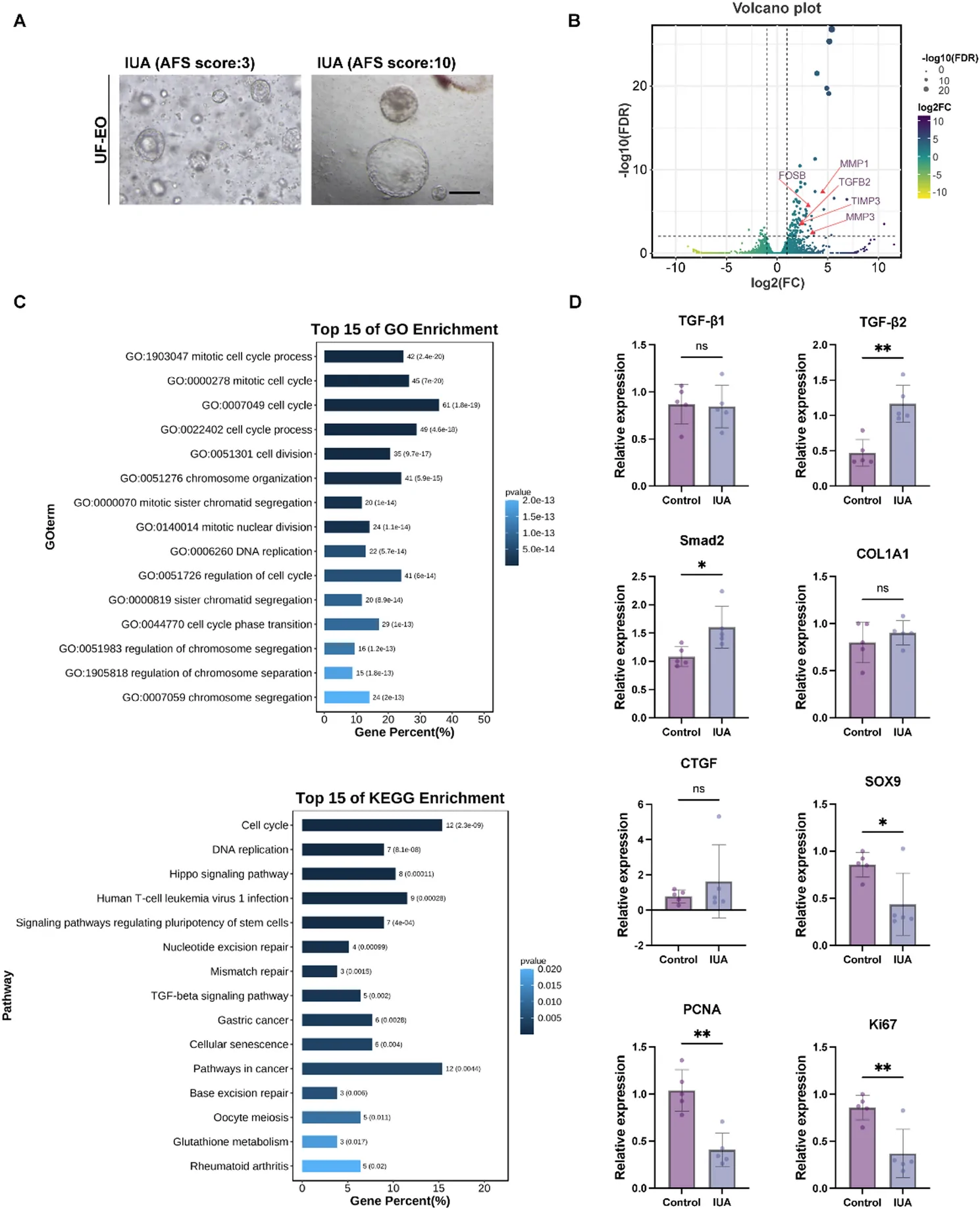
Analysis of hormone-treated UF-EOs derived from both control and IUA group patients. **A** Bright-field microscopy images of UF-EOs derived from IUA patients. **B** A volcano plot depicting differentially expressed genes (DEGs) (absolute fold change > 2 and FDR < 0.05) in UF-EOs between moderate to severe IUA patients (*n* = 3) and controls (*n* = 4). Key DEGs implicated in IUA-related pathological processes are labeled, including upregulated extracellular matrix remodeling genes (MMP1, MMP3, TIMP3), fibrosis regulators (TGFB2), fibroblast activation markers (FOSB). **C** A comprehensive GO and KEGG enrichment analysis of the top 15 pathways was performed on the differentially expressed genes (DEGs) to reveal their biological significance and molecular pathways. (**D**) RT-qPCR validation of TGF-β signaling components, fibrosis markers, stemness, and proliferation markers. Statistical significance was assessed using unpaired t-test, *P*-values are shown on each graph. Data are means ± SEMs. *P < 0.05, **P < 0.01, ***P < 0.001

To establish the clinical applicability of UF-EOs across diverse endometrial disorders, we initially utilized endometrial tissue and uterine lavage fluid from patients diagnosed with repeated implantation failure (RIF) and polycystic ovary syndrome (PCOS) to generate paired organoid lines, thereby demonstrating the feasibility of both culture methods for these patient populations (Supplementary Fig. S1). While these findings validated the versatility of our approach, we prioritized IUA for in-depth investigation due to its unmet need for patient-specific models and the technical inaccessibility of tissue biopsy in advanced cases. RIF and PCOS mechanistic studies were deferred to future work. We therefore focused on intrauterine adhesions (IUA) [19–21], a condition characterized by endometrial fibrosis and compromised regenerative capacity, to establish disease-specific UF-EOs for in-depth mechanistic investigation (Fig. 5A).

In this pilot study, UF-EOs were generated from control subjects without IUA (control group, *n* = 4) and from patients with confirmed IUA (IUA group, *n* = 3, AFS scores 8–10). Organoids were passaged twice to eliminate stromal cell contamination and subjected to hormone treatment to simulate receptive endometrial conditions.

Transcriptomic profiling revealed detectable molecular alterations in IUA-derived UF-EOs. DESeq2 analysis identified 190 differentially expressed genes (DEGs), comprising 162 upregulated and 28 downregulated genes (|fold change| > 2 and FDR < 0.05), as visualized in the volcano plot (Fig. 5B). Key DEGs associated with IUA-related pathological processes were highlighted, including genes involved in extracellular matrix remodeling (MMP1, MMP3, TIMP3) and fibrotic transformation (TGFB2), as well as the fibroblast activation marker FOSB. Given the exploratory nature of this pilot cohort, Gene Ontology enrichment analysis demonstrated that these DEGs were significantly enriched in biological processes related to “cell cycle”, “chromosomal organization”, and “DNA replication” (Fig. 5C). KEGG pathway analysis further revealed significant alterations in “Cell cycle”, “Hippo signaling pathway”, “TGF-beta signaling pathway”, and “cellular senescence” (Fig. 5C), suggesting potential involvement of these pathways in IUA pathogenesis.

Validation by RT-qPCR confirmed key findings from the transcriptomic analysis. While TGF-β1 showed no significant difference between groups, TGF-β2 was significantly upregulated in IUA organoids, accompanied by elevated Smad2 expression (Fig. 5D). Notably, the fibrosis markers COL1A1 and CTGF showed increased expression in IUA samples, though without statistical significance. Conversely, the stemness marker SOX9 was significantly downregulated in IUA organoids, consistent with compromised regenerative capacity. Proliferation markers PCNA and Ki67 were both significantly reduced in IUA-derived UF-EOs, corroborating the transcriptomic enrichment of cell cycle defects.

Collectively, these findings support the potential of UF-EOs as a patient-specific platform for dissecting endometrial pathophysiology and identifying disease-associated molecular signatures.

### UF-EOs as a platform for studying embryo-epithelium interactions

The cystic architecture of endometrial organoids recapitulates key structural features of the uterine epithelium. However, the enclosed luminal configuration and reversed apicobasal polarity inherent to conventional endometrial organoids limit their utility in modeling interactions with embryo surrogates, such as trophoblast models, during implantation [22]. The enclosed luminal compartment presents a technical challenge for studying interactions with trophoblast models. To overcome this limitation, we developed a microinjection approach to deliver HTR8/SVneo trophoblast spheroids directly into the organoid lumen (Supplementary Fig. S2A).

HTR8/SVneo cells were pre-labeled with GFP or mCherry, separately aggregated into spheroids, and microinjected through the Matrigel dome periphery into receptive-phase UF-EOs (EPC condition). This technique enabled delivery of single or multiple trophoblast spheroids into individual organoids while preserving architectural integrity (Supplementary Fig. S2B). Following 24-hour co-culture, confocal z-stack imaging revealed HTR8/SVneo trophoblast spheroids localized within the organoid lumen (Supplementary Fig. S2C). The spheroids were observed in close proximity to the luminal epithelial surface.

## Discussion

Our study introduces uterine lavage fluid as a viable source for generating patient-specific endometrial organoids (UF-EOs). We demonstrate that UF-EOs exhibit glandular architecture, epithelial marker expression, and transcriptome profiles comparable to tissue-derived organoids (T-EOs), with maintained hormone responsiveness. This accessible platform may enable organoid derivation from patient populations previously difficult to study, including individuals with intrauterine adhesions (IUA).

The generation of endometrial organoids has historically relied on invasive tissue acquisition [3, 4]. Although menstrual blood-derived organoids present a significant advance [8–10], their dependence on specific collection devices [23] and the menstrual cycle excludes patients with amenorrhea, oligomenorrhea, or postmenopausal status. Inspired by organoid derivation from other epithelial sources [15], we developed a protocol using uterine lavage fluid. This procedure is non-invasive, integrated into routine gynecological practice, and feasible in patients with compromised endometrial cavities where biopsy is contraindicated.

UF-EOs exhibited robust growth, with self-organization evident within 2–3 days and morphological maturation by days 6–8. Organoids derived from proliferative and secretory phase lavage fluid both exhibited cystic morphologies, and lines were maintained for over 15 passages with successful cryopreservation and recovery. By passages 4–8, expansion rates converged between sources. Matched-patient analysis revealed indistinguishable proliferation rates and viability between UF-EOs and T-EOs. Immunofluorescence analyses confirmed comparable expression of epithelial markers (EPCAM, CK7, and E-cadherin). Transcriptomic analysis showed tight clustering of UF-EOs with matched T-EOs, and RT-qPCR validation confirmed comparable expression of epithelial, proliferation, secretory, and progenitor-associated markers. The absence of differentially expressed genes between UF-EOs and T-EOs (Supplementary Fig. S3) supports that uterine lavage fluid-derived organoids closely resemble the transcriptional identity of tissue-derived organoids.

A critical benchmark for any endometrial model is its capacity to mimic the cyclic hormonal changes of the menstrual cycle [24]. UF-EOs underwent morphological and transcriptional maturation in response to estrogen and progesterone, exhibiting secretory-phase characteristics including glandular folding, subnuclear vacuolization, glycogen accumulation, and upregulation of secretory markers (PAEP, SPP1, 17HSDβ2) [4, 5]. These changes were accompanied by downregulation of proliferation markers (Ki67) [3] and ESR1, with modulation of PGR expression [5, 25–27]. T-EOs exhibited concordant responses, indicating equivalent hormone responsiveness between sources. This hormone-responsiveness supports the physiological relevance of the model. Taking advantage of the defined luminal architecture and preserved hormonal reactivity of the mature organoids, we established a direct microinjection assay to gain access to the luminal compartment. This technique enables the precise delivery of trophoblast spheroids into direct contact with the apical epithelial surface within a confined luminal space. In contrast to conventional co-culture models [28, 29], this configuration more accurately recapitulates the native three-dimensional geometry of embryo–epithelial interactions, while circumventing the technical challenges associated with inducing apical-out polarity [22]. Following 24-hour co-culture, spheroids were localized within the lumen; however, stable adhesion or invasive behavior was not observed. Further optimization of co-culture duration and conditions will be required to assess functional interactions.

The ultimate translational value of a patient-derived model lies in its potential to elucidate disease mechanisms. We specifically applied the UF-EO platform to IUA, a condition characterized by endometrial fibrosis and infertility where epithelial pathophysiology is poorly understood [30, 31]. Notably, we achieved organoid derivation from patients with moderate-to-severe intrauterine adhesions (AFS scores 8–10) at a 60% success rate. To explore the epithelial-intrinsic pathology of IUA, we performed transcriptome sequencing on UF-EOs derived from IUA patients and controls. The upregulation of MMP1, MMP3, and TIMP3 is consistent with dysregulated extracellular matrix remodeling, a hallmark of fibrotic endometrial injury. MMPs are well-established regulators of extracellular matrix remodeling in tissue repair and fibrosis [32, 33]. Concurrently, elevated expression of the fibrogenic cytokine TGFB2, a key inducer of myofibroblast activation and epithelial-mesenchymal transition, suggests that epithelial cells acquire pro-fibrotic phenotypes under fibrotic conditions [34–36]. Furthermore, the emergence of FOSB as a differentially expressed gene aligns with recent single-cell profiling studies identifying FOSB-enriched fibroblast subpopulations in IUA endometrium [37]. Together, these transcriptional changes suggest that IUA-derived organoids may recapitulate key molecular features of epithelial alterations associated with fibrotic microenvironment, supporting their potential utility as patient-specific models for exploring IUA pathogenesis.

To contextualize these gene-level changes within broader biological frameworks, Gene Ontology and KEGG enrichment analyses further revealed significant alterations in biological processes and pathways associated with IUA-related pathology, including “cell cycle”, “chromosomal organization”, “DNA replication”, “Hippo signaling pathway”, “TGF-beta signaling pathway”, and “cellular senescence” (Fig. 5C). Previous studies have established that the TGF-beta signaling pathway, essential for wound healing and fibrosis, can activate fibroblasts, promote epithelial-mesenchymal transition (EMT), and enhance collagen synthesis, contributing to fibrosis [38, 39]. Similarly, the Hippo pathway has been implicated in IUA by regulating cell proliferation and apoptosis, and may contribute to EMT-associated phenotypes in epithelial cells. The interaction between the Hippo pathway and the TGF-β axes creates a regulatory network that influences endometrial fibrosis and IUA progression [40]. RT-qPCR validation confirmed significant upregulation of TGF-β2 and Smad2 in IUA organoids. TGF-β2 and Smad2 have been implicated in fibrotic processes through extracellular matrix deposition and myofibroblast activation [41–43]. The fibrosis markers COL1A1 [36, 39, 44] and CTGF [45] showed increased expression in IUA samples, though without statistical significance. The observed downregulation of SOX9, a marker of endometrial epithelial progenitors [4, 46, 47], suggests compromised regenerative capacity in IUA epithelium. Reduced expression of proliferation markers PCNA [48, 49] and Ki67 corroborates the transcriptomic indication of cell cycle dysregulation.

While our study introduces a promising platform, several limitations should be acknowledged. First, lavage fluid yields lower initial cell numbers than tissue biopsy, which may account for reduced derivation efficiency in severe IUA cases. Optimization of collection volume or processing protocols could improve yield. Second, the current model primarily represents the epithelial compartment, and the lack of stromal, vascular, and immune components may influence the interpretation of disease-associated molecular signatures. Integration of these components would enhance physiological relevance. Third, the IUA cohort was limited in size, and validation in larger, independent populations is warranted to confirm the identified molecular signatures. Fourth, the microinjection model requires further development to demonstrate functional trophoblast-epithelium interactions. Despite these limitations, the UF-EO platform opens multiple avenues for future research, including longitudinal studies of endometrial receptivity across menstrual cycles or treatment interventions [50], high-throughput drug screening for conditions like IUA or endometriosis, and the exploration of personalized organoid-based regenerative therapies [51–53].

In conclusion, UF-EOs closely recapitulate the molecular and functional characteristics of native endometrial epithelium, with hormone responsiveness comparable to organoids derived from endometrial tissue. This non-invasive platform enables patient-specific modeling of implantation disorders and endometrial pathologies, providing a versatile tool for mechanistic research, drug screening, and regenerative medicine applications.

## Supplementary Information

Below is the link to the electronic supplementary material.

Supplementary Material 1.

Supplementary Material 2.

Supplementary Material 3.

Supplementary Material 4.

## Data Availability

The sources of the data and materials used in this study are detailed within the manuscript. Additional information is available upon request from the corresponding author.

## Abbreviations

cAMP: Cyclic Adenosine Monophosphate
CK7: Cytokeratin 7
DMEM/F12: Dulbecco’s Modified Eagle Medium/Nutrient Mixture F-12
E2: Estradiol
EGF: Epidermal growth factor
FBS: Fetal bovine serum
H&E: Hematoxylin and eosin
IF: Immunofluorescence
IUA: Intrauterine adhesion
MPA: Medroxyprogesterone acetate
PAS: Periodic Acid-Schiff
PCOS: Polycystic ovary syndrome
RT-qPCR: Quantitative reverse transcription polymerase chain reaction
RIF: Recurrent implantation failure
RNA-Seq: RNA Sequencing
T-EOs: Endometrial organoids derived from endometrial tissue
TEM: Transmission electron microscopy
UF-EOs: Endometrial organoids derived from uterine lavage fluid
